# Designed α-sheet peptides disrupt uropathogenic *E. coli* biofilms rendering bacteria susceptible to antibiotics and immune cells

**DOI:** 10.1038/s41598-023-36343-6

**Published:** 2023-06-07

**Authors:** Alissa Bleem, Tatum Prosswimmer, Ruying Chen, Thomas F. Hady, Jinzheng Li, James D. Bryers, Valerie Daggett

**Affiliations:** 1 Department of Bioengineering, University of Washington, Seattle, WA 98195 USA; 2 Molecular Engineering Program, University of Washington, Seattle, WA 98195 USA; 3 Department of Surgery and Center for Lung Biology, University of Washington, Seattle, WA 98109 USA; 4 Department of Biochemistry, University of Washington, Seattle, WA 98195 USA

**Keywords:** Molecular conformation, Structural biology

## Abstract

Uropathogenic *Escherichia coli* account for the largest proportion of nosocomial infections in the United States. Nosocomial infections are a major source of increased costs and treatment complications. Many infections are biofilm associated, rendering antibiotic treatments ineffective or cause additional complications (e.g., microbiome depletion). This work presents a potentially complementary non-antibiotic strategy to fight nosocomial infections by inhibiting the formation of amyloid fibrils, a proteinaceous structural reinforcement known as curli in *E. coli* biofilms. Despite extensive characterization of the fibrils themselves and their associated secretion system, mechanistic details of curli assembly in vivo remain unclear. We hypothesized that, like other amyloid fibrils, curli polymerization involves a unique secondary structure termed “α-sheet”. Biophysical studies herein confirmed the presence of α-sheet structure in prefibrillar species of CsgA, the major component of curli, as it aggregated. Binding of synthetic α-sheet peptides to the soluble α-sheet prefibrillar species inhibited CsgA aggregation in vitro and suppressed amyloid fibril formation in biofilms. Application of synthetic α-sheet peptides also enhanced antibiotic susceptibility and dispersed biofilm-resident bacteria for improved uptake by phagocytic cells. The ability of synthetic α-sheet peptides to reduce biofilm formation, improve antibiotic susceptibility, and enhance clearance by macrophages has broad implications for combating biofilm-associated infections.

## Introduction

Short- and long-term medical device implantation increases the risk of infection, particularly for individuals who are compromised by pre-existing injury or illness. Many hospital-acquired infections are associated with the very devices required to provide life-sustaining care. Catheter-associated urinary tract infections account for nearly 40% of all such cases and resulted in an estimated $1.7 billion economic burden in the United States in 2016^1,2^. The complicated nature of these infections, compounded by their association with other risk factors, makes them a common source of secondary sepsis in hospitalized patients^3^. Uropathogenic *Escherichia coli* are the primary causative agents of catheter-associated infections^1^. Urethral catheters inoculate *E. coli* into the bladder and promote colonization by providing a surface for bacterial adhesion and mucosal irritation^4^. After initial attachment via pili and other adhesins, *E. coli* multiply and undergo changes in morphology and signaling (i.e. quorum sensing) to form biofilms, which promote epithelial damage and ultimately lead to renal or even systemic infection^3,5^.

Biofilm formation complicates treatment because biofilm-associated bacteria encase themselves in an extracellular matrix comprised of secreted proteins, polysaccharides, and DNA. This macromolecular scaffold resists antibiotic penetration, promotes unique nutrient exchange pathways, enables adaptive stress responses, allows differentiation of highly protected “persister” cells, and shields constituent microorganisms from the host immune system^6,7^. Current treatments to eradicate biofilm infections typically involve intense antibiotic administration, but these approaches can result in long-term disruption of the host microbiota as well as emergence of multi-drug resistant organisms^8^. Thus, new antimicrobial strategies to “disarm” the infection by targeting biofilm-specific aspects of virulence are being pursued here.

*E. coli* produce adhesive amyloid fibers called curli that serve as scaffolds to stabilize the biofilm and facilitate adhesion to inert surfaces and neighboring host cells^9^. The curli-specific genes are encoded in two divergently transcribed operons—*csgBAC* and *csgDEFG*—and their expression is subject to highly complex and extensive regulation^10,11^. The major component of curli fibrils is CsgA, a 13 kDa protein comprised of five imperfect sequence repeats, with high conservation of serine, glutamine, and asparagine residues^12^. The assembly of functional amyloids is assisted by a “nucleator” protein, CsgB, which contains an amyloidogenic domain to template rapid fibril polymerization on the exterior of the cell^13^. The remaining proteins in the curli expression system serve as outer membrane pores for translocation of amyloid monomers (CsgG), chaperones to prevent premature polymerization of CsgA in the periplasm (CsgC), or additional processors and regulators (CsgD-F)^10,14–20^ (SI Appendix, Fig. S1). As fully assembled amyloid fibrils, curli play a crucial role in uropathogenesis by enabling adhesion, colonization, activation of host immunity, and eventual sepsis^21–24^. Indeed, the *csgA* gene, which encodes the major curlin subunit, is highly conserved among clinical isolates^25–28^. Thus, curli represent an essential component of *E. coli* biofilm virulence as well as an excellent target for therapeutic intervention.

The curli biogenesis system in *E. coli* is remarkably adept in its ability to restrict amyloid formation to the cell surface, minimizing the risks of self-toxicity through accumulation of intracellular aggregates. The curli fibril structure is also remarkably similar to the amyloid fibril deposits (plaques) found in human diseases such as Alzheimer’s disease and Parkinson’s disease. However, little is known about how curli monomers fold from their soluble, monomeric form into aggregation-competent conformations and finally insoluble, β-sheet-rich amyloid fibrils upon reaching the extracellular space. In human amyloid diseases, amyloid-related toxicity is linked to the soluble oligomers formed during aggregation, while the mature fibrils are relatively inert^29^. The heterogeneous, dynamic nature of the oligomeric species has hindered their structural characterization at high resolution, but molecular dynamics (MD) simulations can help circumvent these problems. In the process of characterizing conformational changes associated with the earliest steps in amyloidogenesis by MD, we identified a novel secondary structure*—*α*-sheet*. This structure is formed by a variety of mammalian and bacterial amyloid proteins and peptides when they are simulated under amyloidogenic conditions^30,31^. The α-sheet is similar to the more conventional β-sheet backbone conformation, but it is typified by the regular alternation of consecutive residue (ϕ and ψ) angles in the α_L_ and α_R_ local helical conformations, resulting in an extended chain with polar alignment of the main-chain carbonyl groups on one side of the sheet and amide hydrogens on the other^32,33^.

We have designed synthetic peptides that stably adopt α-sheet structure complementary to the α-sheet structure observed in MD simulations of amyloidogenic proteins^32–35^. These designs are complementary to the α-sheet observed in the simulations to provide selective binding to the early α-sheet oligomers during aggregation. The designs use a templated structure with alternating l- and d-amino acids in two strands connected by a turn to form an α-sheet hairpin. The structure of these designs has been confirmed by a variety of biophysical techniques, including a high-resolution atomistic NMR structure^32,34–37^. Additionally, we have shown that these synthetic peptides (denoted as AP#, and non-α-sheet peptides and P#) inhibit the aggregation of three different mammalian disease targets by preferentially binding the toxic soluble oligomers: β-amyloid (Aβ; Alzheimer’s disease), islet amyloid polypeptide (Type 2 diabetes), and transthyretin (systemic amyloidosis)^34–36^. Synthetic α-sheet peptides also exhibit anti-biofilm activity against two different Gram-positive bacteria that produce functional amyloids as a matrix scaffold material^37,38^. These results support our hypothesis that the α-sheet structure is universally adopted during amyloidogenesis and is associated with toxicity^39^.

In this study, we extend the α-sheet hypothesis to the clinically important Gram-negative *E. coli* bacteria. Synthetic, designed α-sheet peptides inhibited amyloid formation in developing biofilm cultures in a dose-dependent manner, resulting in less robust biofilms. The presence of α-sheet structure during formation of the amyloid fibrils was then confirmed by tracking the structural characteristics of CsgA as it aggregated. Moreover, CsgA fibril formation was selectively inhibited by our α-sheet designs. Finally, biofilms grown in the presence of synthetic α-sheet peptides exhibited enhanced antibiotic susceptibility and immune cell clearance. These results elucidate previously undiscovered mechanisms of curli fibril formation as CsgA passes through α-sheet structure, which can be targeted by de novo designed α-sheet peptides. Furthermore, a variety of experiments utilizing different α-sheet peptides show that their inhibitory effect is not limited to a particular sequence. These unique antimicrobial compounds leverage a novel method of action to tackle biofilm recalcitrance.

## Results

### Designed α-sheet peptides inhibit biofilm formation by specifically targeting curli amyloid fibril formation

**Figure 1:**
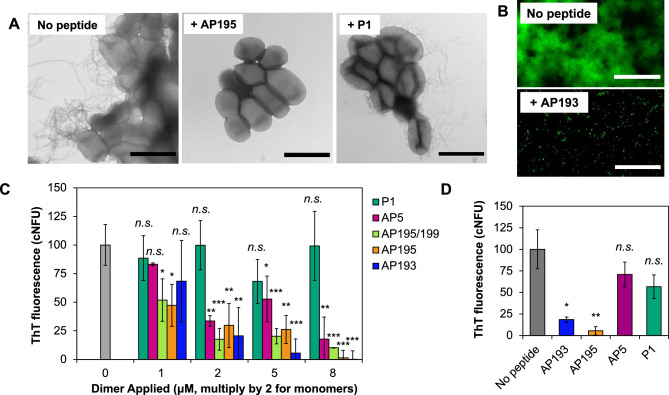
Synthetic α-sheet peptides inhibit curli amyloid formation in biofilms in situ. (**A**) TEM images reveal extensive curli formation in peptide-free biofilms and biofilms grown in the presence of P1, but not those grown in the presence of AP195 (Scale bars = 2 μm). P1 and AP195 were added at equimolar concentrations. AP195 is an α-sheet peptide and P1 is a random coil control. (**B**) Green fluorescent (UTI89 SLC-719) biofilms exhibited far less adhesion to glass slides when grown in the presence of 8 μM (9.6 μg) AP193 (scale bars = 50 μm). (**C**) AP193, AP195, AP195/199, and AP5 caused a dose-dependent reduction in amyloid content in UTI89 biofilms, as measured by ThT fluorescence. The concentrations are provided for the dimers, but the P1 and AP5 monomers were double that concentration to provide the same number of monomer units for comparison of the designs (ex. 5 μM applied AP5 was actually 10 μM). *cNFU* corrected, normalized fluorescence units, where normalized signals were corrected by the nonspecific ThT fluorescence of *E. coli* UTI89 Δ*csgA* biofilms. (**D**) Synthetic α-sheet peptides AP193 and AP195 decreased the ThT fluorescence of *E. coli* GERB319 biofilms, a clinical UTI isolate with resistance to gentamicin and ciprofloxacin (the dimeric peptides were added at 8 μM and AP5 and P1 were 16 μM to provide the same monomer equivalents). The unstructured control peptide P1 had no effect when applied at the same concentration. For panels (**C**) and (**D**), error bars represent the standard deviation from the mean of at least three replicates, and *p*-values are indicated as follows: *p < 0.05, **p < 0.01, ***p < 0.001, and ^n.s.^*p* ≥ 0.05.

To test the effect of α-sheet peptides on amyloid formation in live bacteria, biofilms of the urinary cystitis *E. coli* isolate UTI89 (hereafter, UTI89) were cultivated under conditions known to produce curli biogenesis^40^. Transmission electron microscopy (TEM) was employed to visualize the effect of α-sheet designs on amyloid formation. Dense curli amyloid fibrils were evident in the bacteria without treatment or with administration of a random coil peptide control, P1 (Fig. 1A). In contrast, fibrils were absent in biofilms grown in the presence of an α-sheet peptide design, as shown for AP195 (Fig. 1A).

To investigate the effect of the amyloid fibrils on the strength of the biofilms, we investigated surface adhesion of UTI89. The green-fluorescent strain UTI89 SLC-719, which constitutively expresses vsfGFP-9, was employed for this purpose. Peptide-treated biofilms displayed a more dispersed and soluble phenotype, with far less biomass adhered to glass slides compared with untreated bacteria (Fig. 1B).

In *E. coli* biofilms, curli monomers aggregate to form large, β-sheet-rich amyloid fibrils upon reaching the extracellular space^9,10^. We hypothesized that populations of transient α-sheet oligomers arise during this transition, and that these structures serve as targets for synthetic α-sheet peptide inhibitors to suppress fibril formation. Varying doses of different synthetic α-sheet peptides (AP193, AP195, AP195/199, or AP5) or an unstructured control peptide (P1) were added to replicate cultures at the time of inoculation. AP5 is a 23-residue, monomeric α-sheet hairpin. AP195 and AP193 are homodimers consisting of two identical α-sheet hairpins, and AP195/199 is a heterodimer consisting of one AP195 monomer and one AP199 monomer (SI Table 1; SI Materials and Methods). After 48 h of growth, the biofilms were rinsed, homogenized, and stained with the amyloid dye Thioflavin T (ThT), which fluoresces upon binding β-sheet fibrils and serves as a reporter of amyloid fibril content^41^. ThT also binds nonspecifically to the bacterial cell surface^37^, so biofilms of a UTI89 Δ*csgA* knockout strain were grown in parallel to provide an estimate of nonspecific ThT fluorescence (AP5 is provided as an example in SI Appendix, Fig. S2). This nonspecific signal was subtracted from the UTI89 signals to produce the corrected fluorescence values (cNFU) shown in Fig. 1C.

To investigate the applicability of synthetic α-sheet peptides beyond the well-characterized UTI89, we obtained *E. coli* isolates from pediatric patients who had presented with antibiotic-resistant urinary tract infections. PCR confirmed the presence of the *csgA* gene in all isolates, but only those that exhibited curliated, “rdar” colony morphotypes^42^ on YESCA + Congo Red agar were selected for further characterization (Supporting Information (SI) Appendix, Fig. S3). As with UTI89, AP193 and AP195 caused a significant decrease in ThT fluorescence of the gentamicin/ciprofloxacin-resistant strain GERB319 (Fig. 1D), while the random coil control peptide had no effect. The fact that our α-sheet designs inhibit amyloid formation in these other antibiotic-resistant clinical strains highlights the potential broad antimicrobial utility of synthetic α-sheet peptides.

### CsgA passes through α-sheet secondary structure on the pathway to amyloid formation

The synthetic α-sheet peptides reduced biofilm formation and stability by inhibiting assembly of curli amyloid fibrils in UTI89, but they did not affect cellular growth or development of UTI89 Δ*csgA* biofilms (SI Appendix, Fig. S2). The UTI89 Δ*csgA* strain forms biofilms, but they are less robust in the absence of the amyloid fibrils. The mechanism of inhibition was attributed to specific interactions between the synthetic α-sheet peptides and the major curli substituent, CsgA. Kinetic studies of recombinant CsgA aggregation in vitro were coupled with evaluation of structure by circular dichroism (CD) spectroscopy^43^ to determine whether CsgA forms α-sheet structure and aggregates to form soluble oligomers before converting to β-sheet fibrils. The α-sheet secondary structure displays a unique spectral signature by CD, where alternation of subsequent residues between α_L_ and α_R_ backbone conformation leads to a nearly flat CD spectrum produced by cancellation of the alternating polarized light^33^.

**Figure 2:**
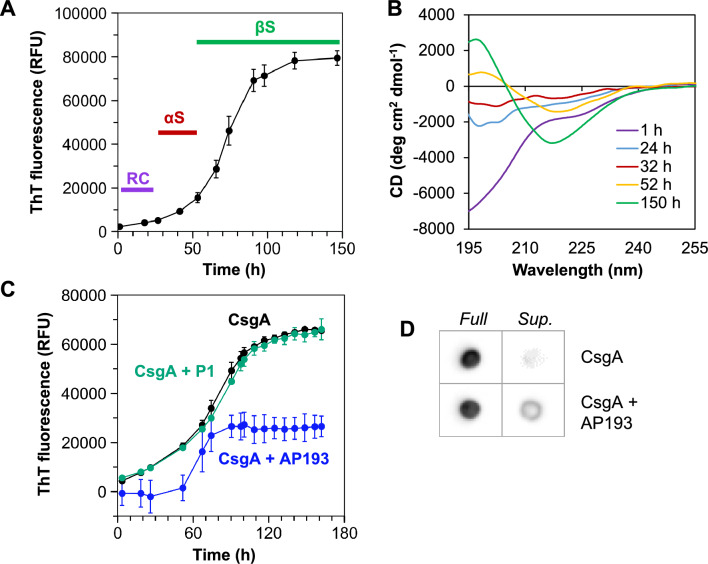
The major curli subunit, CsgA, passes through α-sheet secondary structure on the pathway to amyloid formation. (**A**) Thioflavin T (ThT) fluorescence was used to monitor aggregation of recombinant CsgA at 0.2 mg/mL (~ 14 μM) in 50 mM KPi, pH 6.2. (**B**) Circular dichroism (CD) spectroscopy was used to determine the structure of different conformers populated during aggregation. At the beginning of the assay (purple bars/lines), CsgA was monomeric with random coil (RC) secondary structure. Later in the lag phase (red bars/lines), α-sheet (αS) structure dominated, followed by progression to β-sheet (βS) amyloid fibrils (green bars/lines). (**C**) AP193 (blue line) inhibited CsgA aggregation, while P1 (teal line) did not. In this case, CsgA aggregation was monitored at 10 μM and peptides were applied at 5 μM (AP193, 2:1 molar ratio CsgA:peptide) or 10 μM (P1, 1:1 molar ratio CsgA:peptide). (**D**) Coincubation of CsgA with AP193 suppressed fibril formation and increased the proportion of soluble CsgA in the supernatant (*Sup*.) after centrifugation of endpoint samples. Error bars in (**A**) and (**C**) represent the standard deviation from the mean of at least three replicates.

Recombinant CsgA was purified under denaturing conditions, desalted into potassium phosphate buffer and incubated quiescently at 25 °C. The ThT fluorescence served as a measure of CsgA amyloid fibril formation with respect to time (Fig. 2A). The sigmoidal aggregation kinetics displayed a ~ 40 h lag period followed by a rapid increase in fluorescence, reflecting β-sheet fibril formation, which occurred around 150 h. Different phases of the aggregation process corresponded to different secondary structure adopted by CsgA, as measured by CD. At the beginning of the assay, the ThT fluorescence was low and CsgA adopted random coil secondary structure (Fig. 2A,B, purple lines). Near the end of the lag period, CsgA became enriched in α-sheet secondary structure, as reflected in the relatively flat spectra (Fig. 2A,B, red lines). For reference, similar spectra are obtained for our synthetic α-sheet peptide designs (SI Appendix, Fig. S4). When the ThT fluorescence plateaued, CsgA formed β-sheet structure by CD (Fig. 2A,B, green lines), and insoluble amyloid fibrils were visible to the naked eye.

Additional aggregation assays were carried out to probe the interactions between synthetic α-sheet peptides and their α-sheet-rich CsgA targets. Excess CsgA and synthetic peptides were mixed (2:1 molar ratio CsgA:AP193, or P1). As expected, CsgA incubated alone or with the unstructured control peptide P1 demonstrated a high degree of fibril formation and high ThT fluorescence values (Fig. 2C, black and green lines). In the presence of AP193, however, CsgA fibril formation was reduced (Fig. 2C, blue line). Endpoint samples of CsgA incubated with and without AP193 were also analyzed by immunoblotting to determine the amount of soluble CsgA remaining in the supernatant after high gravity centrifugation (Fig. 2D). CsgA alone converted almost entirely to insoluble fibrils, which were pelleted during centrifugation, resulting in a lack of detectable protein in the supernatant. However, coincubation of CsgA with AP193 led to suppressed fibril formation and increased the proportion of soluble CsgA in the supernatant. These results are in accord with observations in situ, where synthetic α-sheet peptides promoted a more soluble biofilm phenotype through inhibition of curli formation.

### Designed α-sheet peptides increase bacterial susceptibility to antibiotics

Since synthetic α-sheet peptides destabilized the *E. coli* biofilm matrix by interfering with curli assembly, it was hypothesized that they would increase the susceptibility of the bacteria to antibiotics. To test this, UTI89 biofilms were cultivated in the presence of α-sheet peptides. After 42 h of growth, the cultures were supplemented with gentamicin-containing (Gm; 300 μg/mL) or antibiotic-free medium, and biofilms were incubated for an additional 6 h. Biofilms were then harvested and homogenized by ultrasonication, and viable bacteria counts, or colony forming units (CFUs), were determined by the drop plate method^44^. Two designs were investigated: AP90, our benchmark α-sheet design, and AP401^32,34,35,37^. AP90 is a monomeric hairpin peptide with a turn composed of all l-chirality residues. AP401 has 100% sequence identity to AP90, but every residue has the opposite chirality; d-chirality residues make up the turn in AP401.

**Figure 3:**
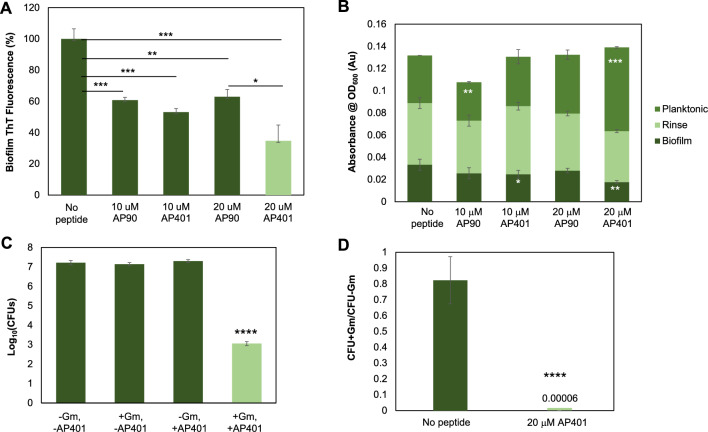
Synthetic α-sheet peptides inhibit fibril formation, shift bacteria from biofilm to planktonic state, and increase susceptibility of UTI89 biofilms to antibiotics. (**A**) Biofilm inhibition was monitored via ThT fluorescence for the monomeric structural isomers, AP90 and AP401. Both peptides provided a significant drop in amyloid formation with AP401 outperforming AP90. (**B**) OD_600_ readings reflect the bacterial content and indicate that AP401 is a more potent inhibitor of curli formation than its structural isomer, AP90. Estimation of bacterial cell counts were performed by collecting and homogenizing the cells during each phase of the ThT assay (planktonic, rinse, biofilm) and the number of cells was estimated according to the optical density of samples at 600 nm. Peptides did not affect growth; instead, they shifted bacteria from the biofilm-associated state to the planktonic state. Error bars indicate the standard deviation from the mean of three replicates. (**C**) Co-administration of 20 μM AP401 and 300 μg/mL gentamicin caused reduction of 10^4^ CFU. (**D**) Co-administration of AP401 and Gm resulted in a 13,333-fold increase in the susceptibility of the bacteria to the antibiotic.

AP401 was a more potent inhibitor of amyloid formation in UTI89 biofilms than AP90 at identical concentrations (Fig. 3A). Amyloid content decreased approximately 65% following incubation with 20 μM AP401, and only 37% with 20 μM AP90 (Fig. 3A). Optical density (OD_600_) measurements, which correlate with the number of bacterial cells, indicated that incubation with 20 μM, or 2 pg/CFU, AP401 reduced biofilm cell density over 45%, while the same concentration of AP90 resulted in only 16% reduction (Fig. 3B). Conversely, the density of planktonic, free-floating cells increased over 75% with AP401, but only 23% with AP90 (Fig. 3B). No statistically significant difference was observed between the total cell densities of each condition, indicating that incubation with AP401 released more cells to the planktonic phase than AP90 without causing cell death. Given that the amino acid sequences of AP90 and AP401 are the same, the elevated potency of AP401 is likely due to the increased stability to proteases imparted by the d-amino acids. The shift from the biofilm fraction to planktonic phase was not limited to AP90 and AP401. Treatment of *E. coli* with dimeric AP designs also shifted cells into the planktonic state and greatly reduced the bacterial content in the biofilm phase (SI Appendix, Fig. S5).

As AP401 was a more potent inhibitor of biofilm formation than AP90, we tested whether the predominantly d-chirality peptide would induce greater UTI89 antibiotic susceptibility than its l-turn counterpart. When UTI89 biofilms were cultivated without any α-sheet peptide, the addition of Gm (300 μg/mL) failed to significantly decrease the number of viable CFUs. Conversely, biofilms grown in the presence of 20 μM AP401 exhibited a CFU reduction of a factor of over 10^4^ when exposed to Gm (Fig. 3C). Importantly, in the absence of antibiotics, biofilms grown with AP401 did not exhibit a CFU reduction compared to their peptide-free counterparts, indicating that α-sheet peptides do not exert a selective growth pressure. Furthermore, AP401 had no effect on antibiotic susceptibility in the *ΔcsgA* strain (SI Appendix, Fig. S6). Comparing the ratio of CFUs with and without antibiotic revealed that incubation with AP401 led to 13,333 times greater antibiotic penetration compared with Gm alone (Fig. 3D).

### Peptide-treated biofilms are more vulnerable to immune clearance

When synthetic α-sheet peptides interfere with curli assembly, the extracellular matrix loses a critical structural reinforcing component, resulting in decreased structural integrity and a reduction in the proportion of biofilm-associated bacteria. In addition to improving antibiotic efficacy, we posited that these structural changes would increase the availability of bacteria for phagocytic clearance by immune cells. As before, biofilms were cultivated in microtiter plates with the synthetic α-sheet peptide AP193 added to the growth medium, and the fluorescent derivative, UTI89 SLC-719^45^, was utilized for these experiments to facilitate visualization. After 48 h, mature biofilms were washed and murine macrophages (RAW 264.7 cells labeled with a red fluorescent tag) were applied to the biofilms for 1 h. Macrophages were separately co-incubated with planktonic bacteria at the same cell ratio as a positive control.

**Figure 4:**
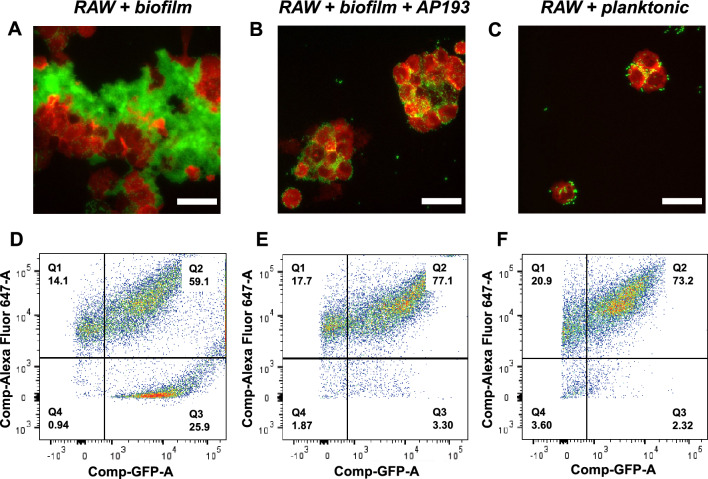
Synthetic α-sheet peptides increase susceptibility of UTI89 bacteria to phagocytosis by macrophages. (**A**–**C**) Fluorescence microscopy (scale bars = 30 μm) images after 1 h coincubation of *E. coli* UTI89 SLC-719 (GFP, green) with RAW 264.7 macrophages (Alexa Fluor 647, red). (**D**) The proportion of Alexa Fluor 647-positive particles that were also GFP-positive for untreated biofilms was 59%, and macrophages had difficulty accessing biofilm-associated bacteria. (**E**) Phagocytosis increased to 77% for biofilms cultivated in the presence of 8 μM AP193. (**F**) The phagocytosis rate for planktonic cells was similar to that of AP193 treated cells, 73%. Improved phagocytosis in the presence of AP193 was attributed to improved biofilm solubility and increased availability of individual bacteria.

When UTI89 and the macrophages were co-incubated, the biofilm matrix shielded the bacteria from the immune cells (Fig. 4A). Addition of macrophages to peptide-treated biofilms resulted in the bacteria being more accessible, and the bacteria were engulfed (Fig. 4B). Planktonic bacteria were co-incubated with the macrophages as a control and they, too, were phagocytosed (Fig. 4C). Thus, curli disruption by synthetic α-sheet peptides induced changes in biofilm architecture, liberating more bacteria for phagocytosis compared to untreated controls.

Analysis by flow cytometry confirmed that the macrophages phagocytosed far more *E. coli* when the biofilms were cultivated in the presence of AP193. Specifically, the proportion of macrophages with internalized bacteria increased from 59% in control biofilms to 77% in peptide-treated biofilms (quadrant 2 in Fig. 4D,E). Accordingly, the proportion of non-phagocytosed bacteria decreased from 26% in control biofilms to 3% in peptide-treated biofilms (quadrant 3 in Fig. 4D,E). Thus, addition of a synthetic α-sheet peptide increased the availability of dissociated bacteria for phagocytosis by RAW cells, resulting in phagocytosis comparable to that of planktonic bacterial controls (Fig. 4F). These flow cytometry measurements correlate with the fluorescence microscopy images of the same samples.

Macrophages were also examined for canonical pro-inflammatory (M1) polarization markers. Incubation of macrophages with synthetic α-sheet peptides caused a downregulation of iNOS transcription but an upregulation of CD86 transcription when measured by RT-qPCR (SI Appendix, Fig. S7). Therefore, increased phagocytosis was largely attributed to changes in biofilm phenotype without global activation of a macrophage pro-inflammatory response. TNFα transcription was also upregulated in macrophages incubated with synthetic α-sheet peptides (SI Appendix, Fig. S7), perhaps because phagocytosis requires membrane exocytosis from organelles that also partake in TNF secretion^46^. These findings suggest that synthetic α-sheet peptides induce meaningful changes in biofilm morphology with implications for infection clearance.

## Discussion

The global threat of antibiotic resistance, coupled with the prevalence of biofilm infections, demands new strategies that specifically target biofilm formation and persistence^47–50^. Current therapies often take one of two approaches to address this problem: (1) physical interventions that mechanically disrupt and remove the biofilm (e.g., wound irrigation) or (2) chemical tactics that systemically target biofilms or modified implant surfaces that are decorated with or release antibiotics or antimicrobial agents^50^. While these strategies have demonstrated some efficacy in the clinic, they risk dislodging a high load of bacteria for downstream colonization, furthering reliance on antibiotics. Recent efforts have shifted to target regulatory and structural aspects of biofilm architecture, with the goal of keeping bacteria in the planktonic state and thus more susceptible to clearance^51–53^. Polysaccharides and nucleic acids serve as important matrix scaffold materials in many clinical pathogens, but in *E. coli*, curli fibrils are one of the most conserved extracellular structures and a widespread contributor to biofilm recalcitrance^25^. Therefore, amyloid fibrils represent a promising target to negate uropathogenic biofilm formation while avoiding selective pressure for antibiotic resistance. Synthetic α-sheet peptides bind and sequester α-sheet-rich oligomers formed on the pathway to amyloid formation in a variety of systems, both mammalian and bacterial^34–38^. This study illustrates the utility of synthetic α-sheet peptides in a new system, curliated *E. coli* biofilms.

*E. coli* strain UTI89 produces large quantities of curli that reinforce the biofilm with thick, rigid pellicles that resist dispersion^54^. Conversely, biofilms grown in the presence of all of the synthetic α-sheet peptides presented here, including monomers and dimers with different sequences, displayed a highly soluble phenotype with scant adhesion to surfaces, no fibril structures visible under TEM, and largely planktonic bacterial populations. Indeed, peptide-treated biofilms displayed amyloid fluorescence signals as low as those for curli-deficient Δ*csgA* biofilms, indicating that synthetic α-sheet peptides specifically inhibit curli formation. These dramatic changes in biofilm structure were achieved without reduction in bacterial viability, decreasing the potential for resistance mutations. Furthermore, curli suppression required low doses of peptide. Synthetic α-sheet peptides exerted an effect at concentrations as low as 1 μM and completely abrogated curli formation at 8 μM—an order of magnitude lower than small-molecule inhibitors previously studied in this system^54^ (125 μM for FN075). Additionally, inhibition was achieved at low ratios of peptide to bacteria, with 8 μM of AP193 dimer or AP195 dimer equating to ~ 0.3 pg peptide per bacterium by the end of the 48 h incubation period.

The dimeric α-sheet peptide designs—AP193, AP195, and AP195/199—were particularly effective in reducing the amyloid content of UTI89 biofilms. The efficacy of these peptides stems from the covalent linkage of two α-sheet hairpins to improve the avidity for binding to the α-sheet oligomers formed en route to the curli fibrils. In addition, surprisingly, our monomeric benchmark design, AP90, was not as effective as the same sequence with reversed chirality, AP401. Inhibition of fibril formation and destabilization of *S. aureus* biofilms was essentially the same with these two peptides^37^. The effect of reversing the chirality of the sequence results in d-amino acids in the turn of the hairpin in AP401, which protects the peptide from proteolysis, suggesting that *E. coli* may have a protease targeting the turn, while *S. aureus* does not.

The activity of these different designed peptides in situ suggested a broad curli inhibition effect that was subsequently extended to more complex scenarios. As in the fluorescence assay for biofilm amyloid formation, addition of α-sheet peptides increased the antibiotic susceptibility of curliated UTI89 biofilms to susceptibility levels observed in curli-deficient Δ*csgA* biofilms. Biofilms with robust extracellular matrices show increased tolerance to antibiotic clearance^55^, so disrupting the matrix scaffold with synthetic α-sheet peptides has important implications for sustaining the efficacy of existing antibiotic drugs and slowing the spread of resistance. The value of suppressing curli formation was further demonstrated in a second test, in which AP193 rendered biofilms more vulnerable to immune clearance by murine macrophages. Notably, redesign to prevent proteolytic cleavage with d-chirality amino acids in its hairpin turn, AP401, exhibited the most potent biofilm inhibition and increased antibiotic susceptibility. The inclusion of d-amino acids in the turn of the α-sheet hairpin deters proteases, which is likely responsible for the improved performance of AP401 relative to its predominately l-chirality counterpart, AP90. This discovery, coupled with knowledge of the increased potency of dimeric α-sheet peptides, suggest that a d-amino acid dominant dimeric design may provide the most effective approach for inhibiting *E. coli* biofilm formation and combatting bacterial infection in the future.

The specific and potent activity of synthetic α-sheet peptides in uropathogenic *E. coli* biofilms suggests a role for α-sheet structure in curli assembly. The inhibition was attributed to an interaction between the two components: synthetic α-sheet peptides bind α-sheet-rich oligomers that arise during aggregation of CsgA, abrogating their further association into fibrils. To test this hypothesis, a series of in vitro experiments with the major curli fibril constituent, CsgA, were carried out. CsgA polymerizes to form amyloid fibrils with similar properties to those formed by mammalian amyloids, and previous studies with CD spectroscopy demonstrated conversion of CsgA from largely unstructured monomers to β-sheet-rich fibrils^56,57^. Yet, these studies failed to capture secondary structure at intermediate time points, i.e., the lag phase of aggregation with the largest population of oligomers. In this study, secondary structure was tracked by CD throughout the aggregation process, and CsgA converted from its unstructured form to β-sheet-rich amyloid fibrils with α-sheet structure arising in between. The onset of α-sheet structure in CsgA was further confirmed by addition of synthetic α-sheet peptide AP193, which inhibited fibril formation.

Independent studies indicate that biofilm formation represents a widespread mechanism of immune evasion^58–60^, and extracellular matrix components, including amyloid fibrils, provide resistance to phagocytosis. Even though the extent of phagocytosis in untreated biofilms was greater than zero (59% of macrophages were positive for GFP-expressing bacteria), fluorescence microscopy imaging confirmed the lack of accessibility to large biofilm particles; macrophages crowded around the edges of the biofilm but were unable to break off small pieces (Fig. 4A). By interrupting the amyloid-enriched extracellular matrix formation and forcing bacteria to remain in a planktonic state, synthetic α-sheet peptides represent a promising opportunity to re-engage host defenses against *E. coli*.

## Concluding remarks

Their resistance to antibiotics and their ability to form biofilms make *E. coli* a major target for molecular engineering approaches to clear these infections, but few current therapies suppress biofilm-specific aspects of virulence. Here, we describe a strategy to prevent formation of curli amyloid fibrils, an important structural component of the biofilm matrix in clinical *E. coli* isolates. Synthetic α-sheet peptides are complementary to α-sheet secondary structure formed by CsgA as it aggregates, and these synthetic peptides bind α-sheet-rich oligomers and block the formation of amyloid fibrils. Without the amyloid fibrils, the biofilms became structurally compromised and more susceptible to antibiotics and immune clearance. While much remains to be done, the encouraging results presented here have potential implications for our ability to combat biofilm infections more broadly.

## Methods

The *E. coli* strains used in this study are listed in SI Appendix Table 1. Overnight cultures were grown in LB medium for ~ 18 h. α-sheet peptides were synthesized and applied at different concentrations to *E. coli* cultures. Samples were incubated at 26 °C for 48 h. After growth, planktonic cells and medium were removed and biofilms were rinsed with PBS. Planktonic cells were spun down and resuspended in PBS, and the optical density of both planktonic and rinse samples was determined at 600 nm to estimate cell densities. Biofilms were homogenized and amyloid fibril formation was determined using a thioflavin T (ThT) assay adapted for biofilms^37^. This assay was chosen because ThT fluoresces upon binding β-sheet fibrils and is frequently used to measure amyloid fibril content^41^. In the case of antibiotic susceptibility tests, biofilms were cultivated and gentamycin was added to wells 6 h before the end of incubation. After incubation, planktonic cells and medium were removed and biofilms were rinsed once in sterile PBS. Biofilms were then resuspended in sterile PBS, homogenized and then diluted in tenfold increments for CFU plate counts with the drop plate method^44^. A synthetic gene corresponding to the *E. coli* CsgA protein, minus its *sec* signal sequence, was designed and synthesized by GenScript (Piscataway, NJ). The gene was cloned, plasmid transformed, and the protein was expressed and purified. CsgA aggregation was monitored by ThT fluorescence. Details for these and other experiments are provided in the SI Appendix.

## Supplementary Information

Supplementary Information.

## Data Availability

The data generated and analyzed during this study are included in the body of the paper and the Supporting Information. Any additional datasets are available from the corresponding author on reasonable request.

## References

[1] Weiner LM (2016). Antimicrobial-resistant pathogens associated with healthcare-associated infections: Summary of data reported to the National Healthcare Safety Network at the Centers for Disease Control and Prevention, 2011–2014. Infect. Control Hosp. Epidemiol..

[2] Hollenbeak CS, Schilling AL (2018). The attributable cost of catheter-associated urinary tract infections in the United States: A systematic review. Am. J. Infect. Control.

[3] Spaulding C, Hultgren S (2016). Adhesive pili in UTI pathogenesis and drug development. Pathogens.

[4] Jacobsen SM, Stickler DJ, Mobley HLT, Shirtliff ME (2008). Complicated catheter-associated urinary tract infections due to *Escherichia coli* and *Proteus mirabilis*. Clin. Microbiol. Rev..

[5] Flores-Mireles AL, Walker JN, Caparon M, Hultgren SJ (2015). Urinary tract infections: Epidemiology, mechanisms of infection and treatment options. Nat. Rev. Microbiol..

[6] Stewart PS (2002). Mechanisms of antibiotic resistance in bacterial biofilms. Int. J. Med. Microbiol..

[7] Hall-Stoodley L, Costerton JW, Stoodley P (2004). Bacterial biofilms: From the Natural environment to infectious diseases. Nat. Rev. Microbiol..

[8] Hooton TM (2010). Diagnosis, prevention, and treatment of catheter-associated urinary tract infection in adults: 2009 International Clinical Practice Guidelines from the Infectious Diseases Society of America. Clin. Infect. Dis. Off. Publ. Infect. Dis. Soc. Am..

[9] Chapman MR (2002). Role of *Escherichia coli* curli operons in directing amyloid fiber formation. Science.

[10] Evans ML, Chapman MR (2014). Curli biogenesis: Order out of disorder. Biochim. Biophys. Acta BBA Mol. Cell Res..

[11] Hammar M, Arnqvist A, Bian Z, Olsén A, Normark S (1995). Expression of two *csg* operons is required for production of fibronectin- and congo red-binding curli polymers in *Escherichia coli* K-12. Mol. Microbiol..

[12] Wang X, Chapman MR (2008). Sequence determinants of bacterial amyloid formation. J. Mol. Biol..

[13] Hammer ND, Schmidt JC, Chapman MR (2007). The curli nucleator protein, CsgB, contains an amyloidogenic domain that directs CsgA polymerization. Proc. Natl. Acad. Sci. U.S.A..

[14] Brombacher E (2003). The curli biosynthesis regulator CsgD co-ordinates the expression of both positive and negative determinants for biofilm formation in *Escherichia coli*. Microbiology.

[15] Evans ML (2015). The bacterial curli system possesses a potent and selective inhibitor of amyloid formation. Mol. Cell.

[16] Goyal P (2014). Structural and mechanistic insights into the bacterial amyloid secretion channel CsgG. Nature.

[17] Schubeis T (2018). Structural and functional characterization of the Curli adaptor protein CsgF. FEBS Lett..

[18] Newman SL, Will WR, Libby SJ, Fang FC (2018). The curli regulator CsgD mediates stationary phase counter-silencing of *csgBA* in *Salmonella typhimurium*. Mol. Microbiol..

[19] Taylor JD (2016). Electrostatically-guided inhibition of Curli amyloid nucleation by the CsgC-like family of chaperones. Sci. Rep..

[20] Klein RD (2018). Structure–function analysis of the curli accessory protein CsgE defines surfaces essential for coordinating amyloid fiber formation. MBio.

[21] Kai-Larsen Y (2010). Uropathogenic *Escherichia coli* modulates immune responses and its curli fimbriae interact with the antimicrobial peptide LL-37. PLoS Pathog..

[22] Hollenbeck EC (2018). Phosphoethanolamine cellulose enhances curli-mediated adhesion of uropathogenic *Escherichia coli* to bladder epithelial cells. Proc. Natl. Acad. Sci..

[23] Hung C, Marschall J, Burnham C-AD, Byun AS, Henderson JP (2014). The bacterial amyloid curli is associated with urinary source bloodstream infection. PLoS One.

[24] Tükel Ç (2010). Toll-like receptors 1 and 2 cooperatively mediate immune responses to curli, a common amyloid from enterobacterial biofilms: TLR2 interacts with TLR1 to recognize curli. Cell. Microbiol..

[25] Schiebel J (2017). Genotypic and phenotypic characteristics associated with biofilm formation by human clinical *Escherichia coli* isolates of different pathotypes. Appl. Environ. Microbiol..

[26] Frömmel U (2013). Adhesion of human and animal *Escherichia coli* strains in association with their virulence-associated genes and phylogenetic origins. Appl. Environ. Microbiol..

[27] Cordeiro MA, Werle CH, Milanez GP, Yano T (2016). Curli fimbria: An *Escherichia coli* adhesin associated with human cystitis. Braz. J. Microbiol..

[28] Hadjifrangiskou M (2012). Transposon mutagenesis identifies uropathogenic *Escherichia coli* biofilm factors. J. Bacteriol..

[29] Bemporad F, Chiti F (2012). Protein misfolded oligomers: Experimental approaches, mechanism of formation, and structure-toxicity relationships. Chem. Biol..

[30] Armen RS, DeMarco ML, Alonso DOV, Daggett V (2004). Pauling and Corey’s α-pleated sheet structure may define the prefibrillar amyloidogenic intermediate in amyloid disease. Proc. Natl. Acad. Sci..

[31] Armen RS, Alonso DOV, Daggett V (2004). Anatomy of an amyloidogenic intermediate: Conversion of β-Sheet to α-sheet structure in transthyretin at acidic pH. Structure.

[32] Maris NL, Shea D, Bleem A, Bryers JD, Daggett V (2018). Chemical and physical variability in structural isomers of an α-sheet peptide designed to inhibit amyloidogenesis. Biochemistry.

[33] Bi TM, Daggett V (2018). The role of α-sheet in amyloid oligomer aggregation and toxicity. Yale J. Biol. Med..

[34] Hopping G (2014). Designed α-sheet peptides inhibit amyloid formation by targeting toxic oligomers. Elife.

[35] Kellock J, Hopping G, Caughey B, Daggett V (2016). Peptides composed of alternating l- and d-amino acids inhibit amyloidogenesis in three distinct amyloid systems independent of sequence. J. Mol. Biol..

[36] Shea D (2019). α-Sheet secondary structure in amyloid β-peptide drives aggregation and toxicity in Alzheimer’s disease. Proc. Natl. Acad. Sci..

[37] Bleem A, Francisco R, Bryers JD, Daggett V (2017). Designed α-sheet peptides suppress amyloid formation in *Staphylococcus aureus* biofilms. Npj Biofilms Microbiomes.

[38] Paranjapye N, Daggett V (2018). *De Novo* designed α-sheet peptides inhibit functional amyloid formation of *Streptococcus mutans* biofilms. J. Mol. Biol..

[39] Daggett V (2006). Alpha-sheet: The toxic conformer in amyloid diseases?. Acc. Chem. Res..

[40] Lim JY, May JM, Cegelski L (2012). Dimethyl sulfoxide and ethanol elicit increased amyloid biogenesis and amyloid-integrated biofilm formation in *Escherichia coli*. Appl. Environ. Microbiol..

[41] GadeMalmos K (2017). ThT 101: A primer on the use of thioflavin T to investigate amyloid formation. Amyloid Int. J. Exp. Clin. Investig. Off. J. Int. Soc. Amyloidosis.

[42] Römling U (2005). Characterization of the rdar morphotype, a multicellular behaviour in *Enterobacteriaceae*. Cell. Mol. Life Sci..

[43] Greenfield NJ (2007). Using circular dichroism spectra to estimate protein secondary structure. Nat. Protoc..

[44] Herigstad B, Hamilton M, Heersink J (2001). How to optimize the drop plate method for enumerating bacteria. J. Microbiol. Methods.

[45] Eshaghi M, Mehershahi K, Chen S (2016). Brighter fluorescent derivatives of UTI89 utilizing a monomeric vGFP. Pathogens.

[46] Arango Duque G, Descoteaux A (2014). Macrophage cytokines: Involvement in immunity and infectious diseases. Front. Immunol..

[47] Hall CW, Mah T-F (2017). Molecular mechanisms of biofilm-based antibiotic resistance and tolerance in pathogenic bacteria. FEMS Microbiol. Rev..

[48] Totsika M (2017). Disarming pathogens: Benefits and challenges of antimicrobials that target bacterial virulence instead of growth and viability. Future Med. Chem..

[49] Matilla-Cuenca L, Toledo-Arana A, Valle J (2021). Anti-biofilm molecules targeting functional amyloids. Antibiotics.

[50] Koo H, Allan RN, Howlin RP, Stoodley P, Hall-Stoodley L (2017). Targeting microbial biofilms: Current and prospective therapeutic strategies. Nat. Rev. Microbiol..

[51] Christensen LD (2013). Clearance of *Pseudomonas aeruginosa* foreign-body biofilm infections through reduction of the cyclic di-GMP level in the bacteria. Infect. Immun..

[52] Baker P (2016). Exopolysaccharide biosynthetic glycoside hydrolases can be utilized to disrupt and prevent *Pseudomonas aeruginosa* biofilms. Sci. Adv..

[53] Novotny LA, Jurcisek JA, Goodman SD, Bakaletz LO (2016). Monoclonal antibodies against DNA-binding tips of DNABII proteins disrupt biofilms in vitro and induce bacterial clearance in vivo. EBioMedicine.

[54] Cegelski L (2009). Small-molecule inhibitors target *Escherichia coli* amyloid biogenesis and biofilm formation. Nat. Chem. Biol..

[55] Billings N (2013). The extracellular matrix Component Psl provides fast-acting antibiotic defense in *Pseudomonas aeruginosa* biofilms. PLoS Pathog..

[56] Wang X, Smith DR, Jones JW, Chapman MR (2006). *In vitro* polymerization of a functional *Escherichia coli* amyloid protein. J. Biol. Chem..

[57] Dueholm MS (2011). Fibrillation of the major curli subunit CsgA under a wide range of conditions implies a robust design of aggregation. Biochemistry.

[58] Thurlow LR (2011). *Staphylococcus aureus* biofilms prevent macrophage phagocytosis and attenuate inflammation *in vivo*. J. Immunol..

[59] Jones CJ, Wozniak DJ (2017). Psl produced by mucoid *Pseudomonas aeruginosa* contributes to the establishment of biofilms and immune evasion. MBio.

[60] Le KY, Park MD, Otto M (2018). Immune evasion mechanisms of *Staphylococcus epidermidis* biofilm infection. Front. Microbiol..

