# Supplementary material for: Designed α-sheet peptides disrupt uropathogenic E. coli biofilms rendering bacteria susceptible to antibiotics and immune cells
Source: Sci Rep. 2023 Jun 7;13:9272. doi: 10.1038/s41598-023-36343-6 (PMC10247742; doi:10.1038/s41598-023-36343-6)
Supplement: Supplementary file 1 — Supplementary Information. [file 41598_2023_36343_MOESM1_ESM.pdf]

Supplementary information for:

Designed α-sheet peptides disrupt *Escherichia coli* biofilms by inhibiting amyloid fibril formation rendering them more susceptible to antibiotics and immune cells

Alissa Bleem, Tatum Prosswimmer, Ruying Chen, Thomas Hady, Jinzheng Li, James D. Bryers, and Valerie Daggett

Materials and Methods

Table

Figures

Materials and Methods

Peptide synthesis. Synthetic α-sheet peptide inhibitors were designed *in silico* as previously described (34), using backbone dihedral angle constraints derived from MD simulations (60, 61). Briefly, peptides contain two α-strands of seven residues each, with amino acids alternating sequentially between L-conformation and D-conformation in each of the strands. The α-strands are connected by a five residue turn comprised of all L-amino acids, which gives the peptide a hairpin shape. Finally, the tail of each strand consists of a Gly and an Arg residue, followed by acetyl and amide caps at the N- and C-terminus, respectively. Peptides were assembled by solid phase peptide synthesis on Rink amide resin with Fmoc chemistry and HBTU activation. Peptides were cleaved from the resin and side chain deprotected by TFA/TIPS/H2O (95:2.5:2.5) and precipitated by cold ether. Crude peptides were purified to >95% by RP-HPLC using 5 μM C12 or C18 100 Å columns (Phenomenex; Torrance, CA) and atomic masses were confirmed by electrospray mass spectrometry on a Bruker Esquire Ion Trap (Bruker; Billerica, MA). Sequences for the three α-sheet designs described in this study (AP193, AP195, and AP5) as well as the unstructured control (P1) are listed in **SI Table 1**. In the case of AP193 and AP195, two monomers were linked together via their Cys residues to form a homodimer; an oxidation that was carried out by dissolving pure peptide monomer in isopropyl alcohol and diluting to 0.1 mg/mL in 100 mM ammonium carbonate buffer, pH ~10. Peptides were oxidized by air at room temperature with stirring for 24 h prior to a second round of purification and mass spectrometry. The same procedure was applied for AP195/199 dimers, but in this case equimolar amounts of AP195 and AP199 monomer were oxidized to form a heterodimer product. Dimerization was confirmed by measuring the concentration of free thiols (indicative of monomeric peptide) in solution using the Ellman's reagent, high performance liquid chromatography (HPLC), and the mass of the monomeric and dimerized products was verified using mass spectrometry. All peptides were lyophilized after purification and stored at -20 or -80°C until use.

Biofilm culturing and assays. The *E. coli* strains used in this study are listed in **SI Table 1**. Overnight cultures were grown in LB medium (Thermo Scientific; Waltham, MA) for ~18 h. Bacteria were collected by centrifugation at 8000 x*g* for 3 min, resuspended in YESCA broth (53) + 4% v/v DMSO (Corning; Manassas, VA), and diluted to an optical density of 0.1 at 600 nm. Peptide stocks were dissolved in water and concentrations were determined by Nanodrop™ (Thermo Scientific; Waltham, MA). 20 µL of peptide stock and/or sterile ddH2O was added to each well of a sterile 48 well plate such that the final peptide concentration was 0, 2, 4, 10, or 16 µM (0, 1, 2, 5, or 8 µM for dimeric peptides), then 180 µL of diluted bacteria culture was added on top. Plates were covered, sealed in plastic bags, and incubated at 26°C for 48 h. After growth, planktonic cells and medium were removed and biofilms were rinsed once with 250 µL PBS. Planktonic cells were spun down and resuspended in PBS, and the optical density of both planktonic and rinse samples was determined at 600 nm to estimate cell densities. The PBS solution was removed and biofilms were resuspended in 250 µL PBS + 20 µM ThT (Sigma-Aldrich; St. Louis, MO). Biofilms were homogenized by vigorous pipetting (30x per well), 3 min sonication, and 1 min on a plate shaker. 100 µL of each biofilm suspension was then transferred to a black-walled, clear-bottom 96 well plate for measurements in a plate reader (PerkinElmer; Waltham, MA). ThT fluorescence was measured at 438/495 nm as a proxy for amyloid formation, and biofilm absorbance was measured at 600 nm to estimate bacterial cell density. For UTI89 WT, biofilm ThT fluorescence values were normalized to the average value of peptide-free controls, and then the average fluorescence value of UTI89 Δ*csgA* samples was subtracted to account for nonspecific binding. In the case of antibiotic susceptibility tests, biofilms were cultivated in the same manner, but 100 µL YESCA or 100 µL YESCA supplemented with 900 µg/mL gentamicin (Thermo Scientific; Waltham, MA) was added to wells 6 h before the end of incubation. After incubation, planktonic cells and medium were removed and biofilms were rinsed once in sterile PBS. Biofilms were then resuspended in sterile PBS, homogenized by

ultrasonication for 5 s on ice, and then diluted in tenfold increments for CFU plate counts with the drop plate method (44).

CsgA expression and purification. A synthetic gene corresponding to the *E. coli* CsgA protein, minus its *sec* signal sequence, was designed and synthesized by GenScript (Piscataway, NJ). The gene was cloned into the pET30a(+) vector, which added a C-terminal 6x His tag for purification. Plasmids were transformed into *E. coli* BL21 (DE3) cells and protein expression was carried out in 2 L shake flasks at 37°C. Cultures grew to an OD600nm of 0.6-0.8 prior to induction with 1 mM IPTG. After 3-4 hours of additional growth, cells were harvested by centrifugation and resuspended in 30 mL denaturing buffer (8M Gnd-HCl, 50 mM NaPi, pH 8.0) and lysed overnight with stirring at 4°C. Insoluble material was removed by centrifugation at 14,000 x*g* for 30 minutes and 15 mL of supernatant was incubated with 5 mL HisPur Ni-NTA beads (Thermo Fisher; Waltham, MA) for 2 h at room temperature with end-over-end rotation. The beads were then washed twice with denaturing buffer, twice again with denaturing buffer plus 15 mM imidazole, and twice again with denaturing buffer plus 30 mM imidazole. Finally, protein was eluted with denaturing buffer plus 400 mM imidazole. Samples from each step of the purification were precipitated from guanidinium hydrochloride by trichloroacetic acid(62) and analyzed by SDS-PAGE. Purified fractions were of high concentration and purity, so they were not purified further prior to aggregation assays.

Aggregation assays and analysis. Immediately prior to use, protein eluents were thawed and desalted according to the Zeba desalting column protocol (Thermo Fisher) into 50 mM potassium phosphate pH 6.2 and kept on ice. Protein concentration was determined by absorption at 280 nm, and the stock was diluted to a working concentration of 0.2 mg/mL (~14 µM) or 10 µM, depending on the assay. ThT stock was added to a concentration of 20 µM, and then CsgA was aliquotted into black-walled, clear-bottom 96 well plates at 100 µL per well. Plates were incubated in the dark at 25°C without shaking, and fluorescence measurements were taken

every ~12 h on a plate reader (PerkinElmer) with soft motion robotics to avoid agitation. Every well in the plate was read at every timepoint, regardless of the number of wells containing sample, in order to maintain consistent agitation. In the case of peptide inhibition tests, synthetic α-sheet peptide AP193 was prepared as above and added to the incubation mixture at a concentration of 5 μM (2:1 molar ratio CsgA:peptide). Corrected fluorescence values at each timepoint were calculated by subtracting the fluorescence values of peptide alone from those of CsgA plus peptide. For CsgA solubility tests, endpoint samples (~180 h) were removed from the plate and 2 μL was spotted in triplicate on nitrocellulose membranes. Samples were then centrifuged at 20,000 x*g* for 10 min and the supernatants were spotted in the same manner. Membranes were blocked in PBS + 0.05% v/v Tween-20 + 3% w/v BSA (PBS-T/BSA) for 1 h. Primary antibody (polyclonal anti-CsgA, courtesy of Dr. Matthew Chapman, diluted 1:10,000 in PBS-T/BSA) was applied to the membranes for 1 h. Membranes were then washed three times for 5 min each in PBS-T and secondary antibody was added for 45 min (goat anti-rabbit IgG-HRP, diluted 1:10,000 in PBS-T/BSA. Santa Cruz Biotechology; Dallas, TX). Membranes were washed three times again and then developed with Pierce ECL substrate (Thermo Fisher) according to the manufacturer's instructions. Chemiluminescence images were captured on an Azure c300 imaging system (Azure Biosystems; Dublin, CA).

Circular dichroism. At given timepoints, CsgA samples for CD were transferred directly from microtiter incubation plates to a 1 mm quartz cuvette. CD spectra were recorded on a J-720 spectropolarimeter (Jasco, Inc.; Easton, MD) with the following settings: wavelength 260-195 nm, 0.5 nm data pitch, 1 s integration, 2 nm bandwidth, 25°C, and 6 accumulations. Only data points with detector voltages below 600 V were used. All spectra were corrected by a blank spectrum of relevant buffer and smoothed with a Savitsky-Golay filter at window size 35 and polynomial order 2. The same protocol and settings were used for the designed peptides at 25 μM.

Microscopy. For adherence tests, UTI89 WT biofilms were grown in 8 well Chamber Slides (Lab-Tek™, Thermo Fisher) for 48 h in YESCA broth + 4% DMSO. Planktonic cells and medium were removed, biofilms were rinsed once with PBS, and biofilms fixed with 4% paraformaldehyde and stained with SYTO 9 (Thermo Fisher) prior to imaging on a Zeiss Axioscope inverted fluorescence microscope using the GFP filter (Carl Zeiss AG; Oberkochen, Germany). For phagocytosis, samples were prepared as described below for flow cytometry. A portion of each sample was applied to a glass microscope slide using a Cytospin™ centrifuge (Thermo Fisher), and slides were imaged on an Invitrogen (Thermo Fisher) EVOS™ FL upright fluorescent microscope using the GFP and RFP filters. For TEM, UTI89 WT biofilms were cultivated with or without peptide for 48 h in YESCA broth + 4% DMSO. Planktonic cells and medium were removed, biofilms rinsed once with PBS, then gently scraped off plates and deposited into 20 µL of sterile PBS. Copper-formvar grids (200 mesh; Ladd Research; Williston, VT) were glow discharged and 3 µL biofilm samples were applied to grids for 2 min in a humidified chamber. Grids were then rinsed five times by touching the grid on a clean drop of water and blotting dry. Samples were stained with 2% uranyl acetate (centrifuged for 5 min at 6000 rpm before use) for 90 s in a humidified chamber, blotted dry, air dried for 5 min, and vacuum dried for 2 min. Images were collected on a Philips (Amsterdam, Netherlands) CMS100 electron microscope at 8 kV with an Olympus Morada digital camera (Olympus; Center Valley, PA) and iTEM Software Version 5.0 (iTEM; Irvine, CA). All image post-processing and analysis was conducted in the Fiji distribution of ImageJ (63).

Phagocytosis and flow cytometry. Biofilms of green-fluorescent *E. coli* UTI89 SLC-719 were grown in YESCA broth + 4% DMSO for 48 h in 48 well polystyrene plates at 26°C. Planktonic cells and medium were removed and biofilms were washed once with sterile PBS. RAW 264.7 macrophage cells were grown in complete medium (DMEM + 10% fetal bovine serum + 1X penicillin/streptomycin) to passage 12 ± 2, stained with CellTrace™ Red (Thermo Fisher;

Waltham, MA), and resuspended in FACS buffer (PBS + 5% FBS). For coincubation, 250 μL of stained macrophage suspension was applied on top of each biofilm at a MOI of 1:100 (macrophage:bacteria). Planktonic bacteria and macrophages were coincubated separately at the same ratio. Coincubation proceeded for 1 h at 37°C prior to detachment of cells and biofilm by gentle scraping. Suspensions were collected and centrifuged for 5 min at 800 *xg*. The supernatant was aspirated and pellets were resuspended in FACS buffer. 10,000 events per sample gated on single cells were acquired on a BD LSRII flow cytometer (BD Biosciences; Franklin Lakes, NJ) and analyzed using FlowJo software (BD Biosciences). The fraction of macrophage phagocytosing bacteria was calculated as the ratio of AF647/GFP double positive population to AF647 positive population.

RT-qPCR. For analysis of *csgA* expression in *E. coli* UTI89 WT, biofilms were cultivated in 48 well plates with or without 8 μM AP193, as described above. Biofilm-associated bacteria were collected and normalized to a concentration of 10^8 cells/mL. Bacterial mixtures were combined with RNAprotect Bacteria Reagent (Qiagen; Hilden, Germany) and lysed. For analysis of macrophage polarization markers, RAW 264.7 cells were seeded in 24 well plates at ~5 x 10^4 cells per well. After 24 h, the growth medium was aspirated and replaced with media supplemented with synthetic peptides (5 μM AP193, 5 μM AP195, 10 μM AP5, 10 μM P1), or sterile water in the case of controls. The plate was incubated a further 24 h, then cells were trypsinized to detach them from the plate, washed, and resuspended in FACS buffer. For all samples, total RNA was extracted and purified with the RNeasy Mini Kit (Qiagen) according to manufacturer instructions. cDNA was synthesized with iScript RT Supermix (BioRad; Hercules, CA) and quantified with the Qubit system (Thermo Fisher). Each RT-qPCR reaction utilized 10-20 ng of cDNA, 10 μL Power SYBR Green Master Mix (Thermo Fisher), and 0.4 μM each of forward and reverse primers; samples were prepared in duplicate with appropriate housekeeping gene controls and template-free controls (primers in **SI Table 1**). Thermal cycling was carried out

on an Applied Biosystems 7900HT Fast Real-Time PCR system (Thermo Fisher) with 40 cycles of 95°C for 15 s and 60°C for 1 min. Data were analyzed in SDS Software v2.4 (Thermo Fisher).

***Statistics*.** Significance tests for bar plots are based on *p*-values generated by a two-tailed, homoscedastic Student's t-Test, where * indicates $p < 0.05$, ** indicates $p < 0.01$, *** indicates $p < 0.001$, and n.s. indicates $p \geq 0.05$.

SI Table 1. Peptide and primer sequences; bacterial strain descriptions. [a] "AP" refers to "Alternating Peptide", indicating alternating L- and D- amino acid templating. "P" refers to "Peptide", indicating a lack of templating. [b] L- amino acids are displayed in all upper case; D-amino acids are displayed in lower case and underlined. [c] F = forward primer; R = reverse primer. * Indicates housekeeping gene

Peptide sequences

Name[a]	*Sequence[b]*	*Description*	*Source*
P1	Ac-KLKpLLTSENTL-NH2	random coil control	Hopping et al., 2014
AP90	Ac-RGEmNlSwMNEYSGWtMnLkMGR-NH_2	α-sheet monomer	Hopping et al., 2014
AP401	Ac-rGeMnLsWmneysGwTmNlKmGr-NH2	α-sheet monomer	Bleem et al., 2017
AP193	Ac-RGEmNyFwMNEYYGWtMnCkMGR-NH2	homodimeric α-sheet hairpin	Hopping et al., 2014
AP195	Ac-RGNwNeSkMNEYSGWmLmCtMGR-NH2	homodimeric α-sheet hairpin	This work
AP195/199	AP195 + Ac-RGEmNlSwMNEYSGWtMnCkMGR-NH2	heterodimeric α-sheet hairpin	This work
AP5	Ac-RGNwNeSkMNEYSGWmLmLtMGR-NH2	monomeric α-sheet hairpin	Kellock et al., 2016

qPCR primer sequences

Gene	*Oligonucleotide sequence[c], 5'-3'*	*Amplicon size (bp)*	*Source*
iNOS	F: GCCACCTTGGTGAAGGGACT<br>R: ACGTTCTCCGTTCTCTTGCAGT	111	This work
TNFα	F: GTCCCCAAAGGGATGAGAAGT<br>R: TTTGCTACGACGTGGGCTAC	124	This work
CD86	F: AACTTACGGAAGCACCCACG<br>R: CTCCACGGAAACAGCATCTGAG	101	This work
*PPIA**	F: GTCTCCTTCGAGCTGTTTGC<br>R: GCGTGTAAAGTCACCACCCT	150	This work

PCR primer sequences

Gene	*Oligonucleotide sequence[c], 5'-3'*	*Amplicon size (bp)*	*Source*
csgA	F: GGCCCAAATTCTGAGCTGAAC<br>R: ATGAGCGGTCGCGTTGTTA	330	This work

***E. coli* strains**

Strain	*Relevant traits*	*Source*
UTI89	UPEC strain; cystitis isolate	Mulvey et al., 2001
UTI89 Δ*csgA*	UPEC strain; cystitis isolate with chromosomal deletion of *csgA* gene	Cegelski et al., 2009
UTI89 SLC-719	Derivative of UTI89 expressing chromosomal vsfGFP	Eshaghi et al., 2016
GERB 319	Clinical isolate from pediatric UTI; resistant to gentamicin and ciprofloxacin	Evgeni Sokurenko

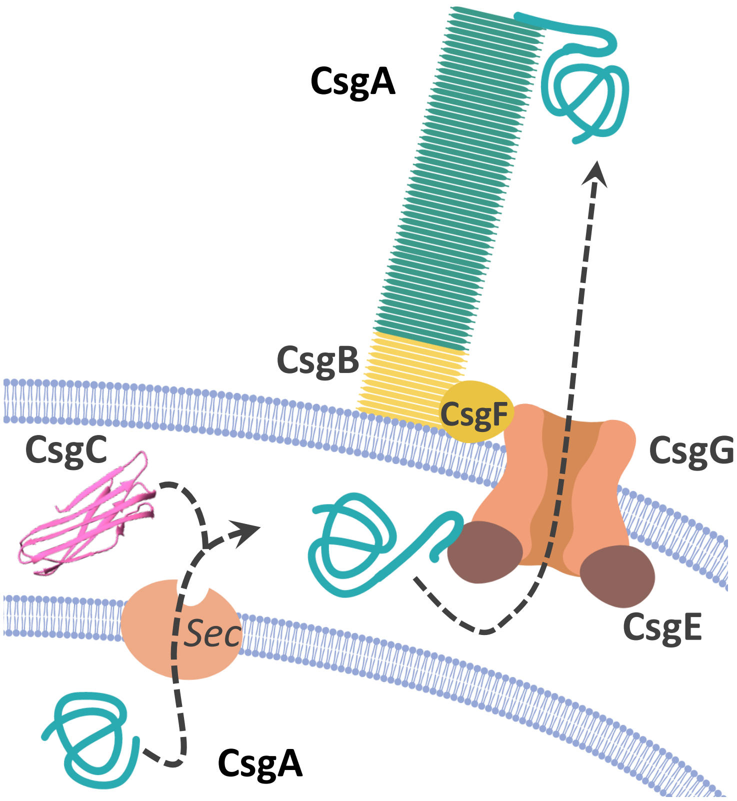

SI Figure S1. *E. coli* produce curli through a coordinated system of proteins. The major curli subunit, CsgA, begins as a soluble monomer in the cytoplasm prior to its export and cleavage by the general secretory (Sec) system. In the periplasm, CsgA interacts with chaperone CsgC and is then exported to the extracellular space by CsgE and CsgG. There, CsgB serves as a template for rapid amyloid fiber formation by CsgA, and CsgF anchors the fibril complex. See references 9 and 10 for further information.

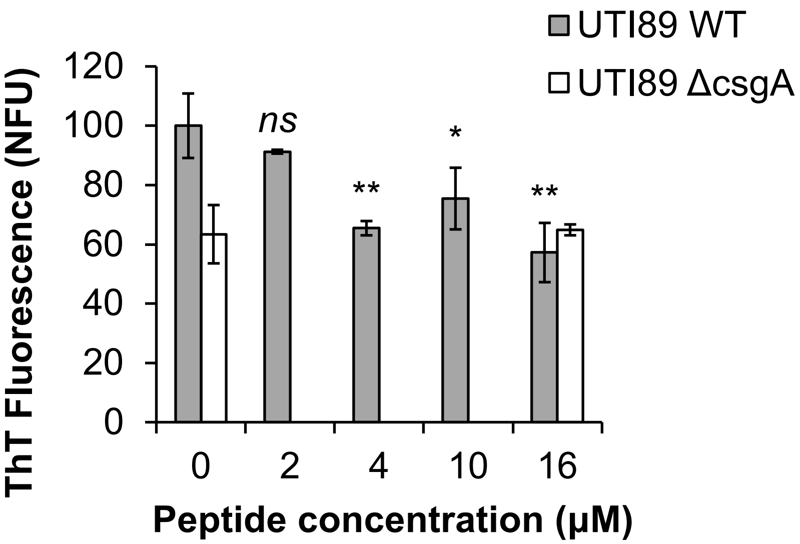

SI Figure S2. Normalized ThT fluorescence values for *E. coli* UTI89 WT biofilms (gray bars) grown in the presence of increasing concentrations of the synthetic α-sheet peptide, AP5. Biofilms of UTI89 Δ*csgA* (white bars) were cultivated in the same experiment to provide an estimate of non-specific ThT fluorescence. This non-specific signal was subtracted from UTI89 WT signals to produce the corrected fluorescence values shown **Figure 1C,D**. Error bars indicate the standard deviation from the mean of three replicates. *ns* = not significant; * $p < 0.05$; ** $p < 0.01$ according to a two-tailed, homoscedastic Student's t-Test.

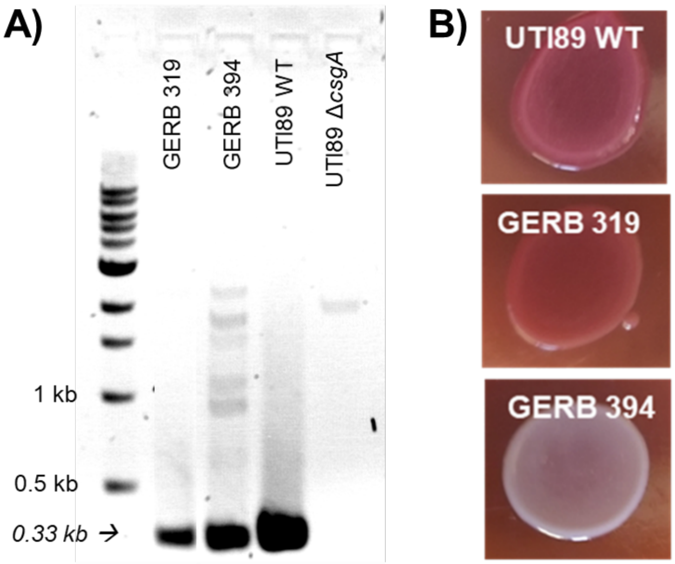

SI Figure S3. The antibiotic-resistant, clinical *E. coli* strain GERB 319 demonstrates a strong curliated phenotype, as demonstrated by the presence of both **(A)** the *csgA* gene (330 bp target amplified by PCR from genomic DNA) and **(B)** “rdar” colony morphotype. Conversely, strain GERB 394 contains the *csgA* gene but lacks the “rdar” morphotype.

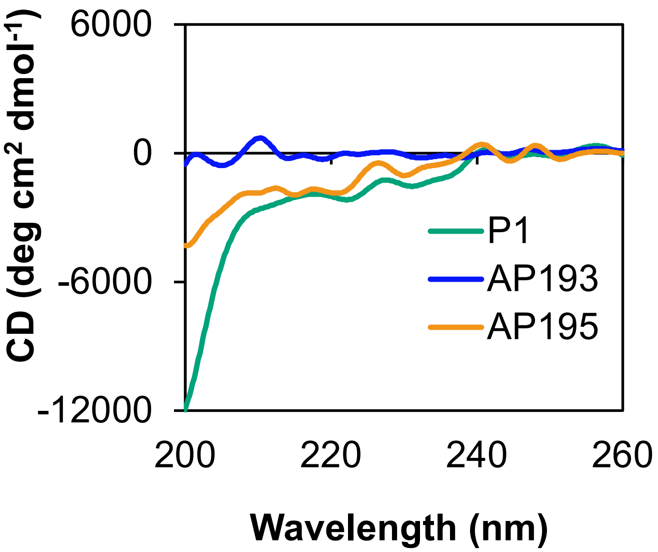

SI Figure S4. Alternating backbone chirality generates uniquely featureless CD spectra (blue and orange lines for dimeric α-sheet peptides AP193 and AP195, respectively) compared to that for a peptide with random coil secondary structure (green line for random coil peptide P1).

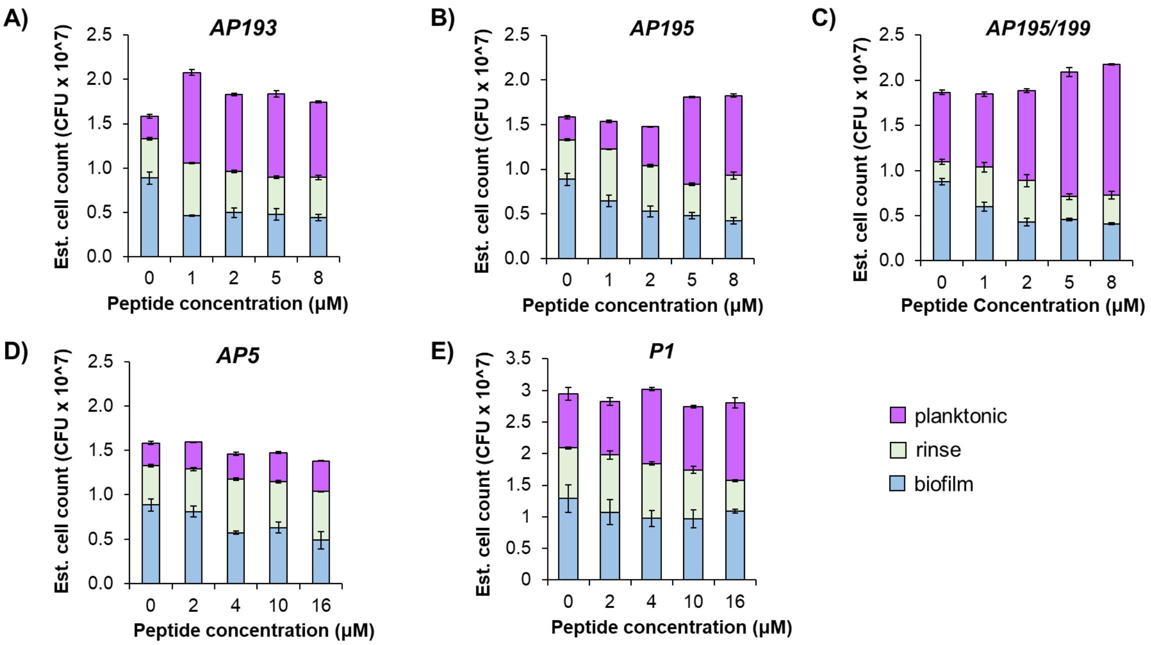

SI Figure S5. Estimation of bacterial cell counts in biofilm ThT assays. Cells were collected and homogenized during each phase of the assay (planktonic, rinse, biofilm) and the number of cells was estimated according to the optical density of samples at 600 nm. Peptides did not affect growth; instead, they shifted bacteria from the biofilm-associated state to the planktonic state. Error bars indicate the standard deviation from the mean of three replicates. AP193 and AP195 are homodimers, AP193/AP195 is a heterodimer, and AP5 and P1 are monomers.

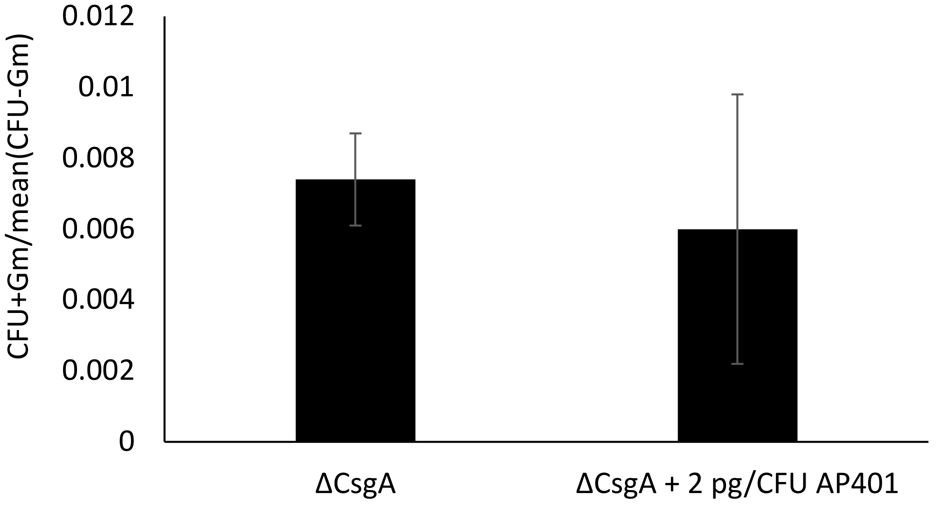

SI Figure S6. AP401 has no effect on antibiotic susceptibility of *E. coli* in CsgA knockout. p-value > 0.05.

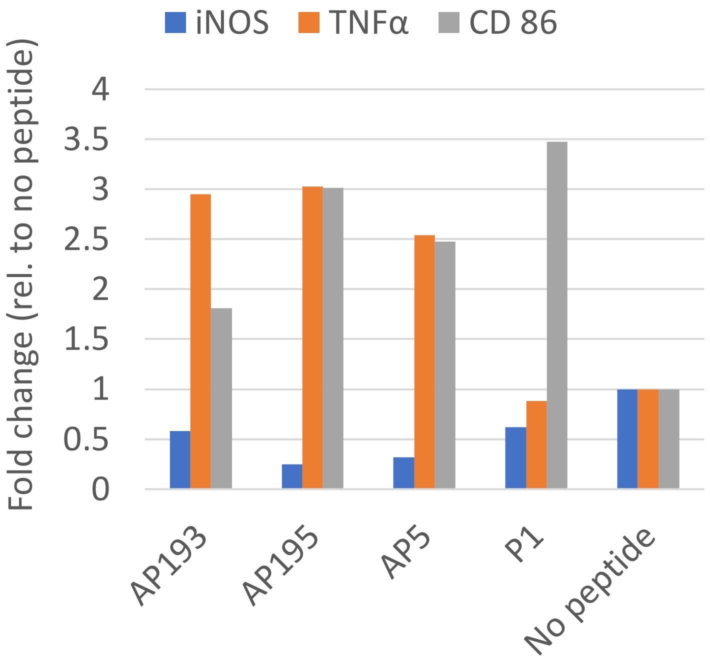

SI Figure S7. Mixed polarization of RAW 264.7 cells in the presence of synthetic α-sheet peptides. Macrophages were grown on 24 well polystyrene tissue culture plates with the indicated peptides for 24 h, and then expression of pro-inflammatory (“M1”) markers was evaluated by RT- qPCR. Expression of TNFα and CD 86 increased compared to peptide-free controls, but expression of iNOS decreased. Data are expressed in terms of a fold change in the relative transcript abundance compared to the housekeeping gene (cyclophilin A; PPIA) control.
